# The central role of mosquito cytochrome P450 CYP6Zs in insecticide detoxification revealed by functional expression and structural modelling

**DOI:** 10.1042/BJ20130577

**Published:** 2013-09-13

**Authors:** Alexia Chandor-Proust, Jaclyn Bibby, Myriam Régent-Kloeckner, Jessica Roux, Emilie Guittard-Crilat, Rodolphe Poupardin, Muhammad Asam Riaz, Mark Paine, Chantal Dauphin-Villemant, Stéphane Reynaud, Jean-Philippe David

**Affiliations:** *Laboratoire d’Ecologie Alpine (LECA), UMR 5553 CNRS, Université de Grenoble, Grenoble 38041, France; †Institute of Integrative Biology, University of Liverpool, Liverpool L69 7ZB, U.K.; ‡Université Pierre et Marie Curie, CNRS, Paris 75005, France; §Liverpool School of Tropical Medicine, Liverpool L3 5QA, U.K.; ∥Department of Agri-Entomology, University College of Agriculture, University of Sargodha, Sargodha, Pakistan; ¶Department of Ecology and Evolution, University of Lausanne, Lausanne 1015, Switzerland

**Keywords:** cytochrome P450 monooxygenase, insecticide, metabolism, mosquito, pyrethroid, recombinant system, resistance, AeCPR, *Aedes aegypti* CPR, CPR, NADPH-cytochrome P450-reductase, Cyt b5, cytochrome b5, 7-OH, 7-hydroxycoumarin, PBA, 3-phenoxybenzoic acid, PBAlc, 3-phenoxybenzoic alcohol, PBAld, 3-phenoxybenzaldehyde, qPCR, quantitative real-time PCR, *R*_t_, retention time, SRS, substrate recognition site, TFA, trifluoroacetic acid

## Abstract

The resistance of mosquitoes to chemical insecticides is threatening vector control programmes worldwide. Cytochrome P450 monooxygenases (CYPs) are known to play a major role in insecticide resistance, allowing resistant insects to metabolize insecticides at a higher rate. Among them, members of the mosquito CYP6Z subfamily, like *Aedes aegypti* CYP6Z8 and its *Anopheles gambiae* orthologue CYP6Z2, have been frequently associated with pyrethroid resistance. However, their role in the pyrethroid degradation pathway remains unclear. In the present study, we created a genetically modified yeast strain overexpressing *Ae. aegypti* cytochrome P450 reductase and CYP6Z8, thereby producing the first mosquito P450–CPR (NADPH-cytochrome P450-reductase) complex in a yeast recombinant system. The results of the present study show that: (i) CYP6Z8 metabolizes PBAlc (3-phenoxybenzoic alcohol) and PBAld (3-phenoxybenzaldehyde), common pyrethroid metabolites produced by carboxylesterases, producing PBA (3-phenoxybenzoic acid); (ii) *CYP6Z8* transcription is induced by PBAlc, PBAld and PBA; (iii) *An. gambiae* CYP6Z2 metabolizes PBAlc and PBAld in the same way; (iv) PBA is the major metabolite produced *in vivo* and is excreted without further modification; and (v) *in silico* modelling of substrate–enzyme interactions supports a similar role of other mosquito CYP6Zs in pyrethroid degradation. By playing a pivotal role in the degradation of pyrethroid insecticides, mosquito CYP6Zs thus represent good targets for mosquito-resistance management strategies.

## Abstract 2

The key role of mosquito cytochrome P450 monooxygenases from the CYP6Z subfamily in the resistance of mosquitoes to insecticides was revealed. By using functional expression and *in silico* modelling, the capacity of CYP6Zs to degrade insecticide metabolites produced by esterase-mediated hydrolysis was demonstrated.

## INTRODUCTION

Mosquitoes transmit numerous parasites and viruses responsible for severe human diseases, such as malaria or dengue. These diseases represent an important burden in tropical and subtropical regions, predominantly affecting developing countries [1]. Indeed, half of the world's population is at risk of malaria, whereas dengue represents a major threat in over 100 countries with more than 2.5 billion people at risk [1].

In the absence of efficient treatments or vaccines, vector control often represents the most effective means for limiting disease transmission [2]. Effective vector control largely relies on the use of insecticides targeting adults or larvae [3] and, because of their high efficiency and cheapness, chemical insecticides remain the first line of defence against mosquitoes when disease prevalence is high. Chemical insecticides used for mosquito control belong to various chemical families, from which pyrethroids are mainly used for impregnating bednets and spraying.

However, resistance of mosquitoes to insecticides is threatening vector control programmes worldwide [4]. Resistance can be the consequence of a mutation of the protein targeted by the insecticide (target-site resistance), a lower penetration or a sequestration of the insecticide, or an increased biodegradation of the insecticide (metabolic resistance) [5,6]. Detoxification enzymes such as cytochrome P450 monooxygenases (P450s or CYPs), GSTs and CCEs (carboxy/choline esterases) are known for their roles in insecticide metabolism in insects [7,8] and their overproduction has been frequently associated with resistance to chemical insecticides in mosquitoes [5,6].

P450s are haem-thiolate-containing enzymes present in almost all organisms and are involved in the metabolism of a wide range of molecules [9]. Most P450s involved in detoxification processes are expressed in the endoplasmic reticulum and catalyse the oxidation of xenobiotics or endogenous compounds in the presence of their obligatory electron donor CPR (NADPH-cytochrome P450-reductase) and sometimes Cyt b5 (cytochrome b5) [10]. Insect P450s are involved in metabolic resistance to various insecticides [7,9,11,12]. In mosquitoes, P450s are encoded by more than 100 *CYP* genes [13,14].

Following the development of transcriptomic tools in mosquitoes [14,15], several P450s overtranscribed in pyrethroid-resistant mosquitoes were identified [5,6]. Some of them have been validated as pyrethroid metabolizers such as *Anopheles gambiae* CYP6M2 and CYP6P3 [16,17], *Anopheles funestus* CYP6P9b [18] and *Aedes aegypti* CYP9J32 [19]. Among mosquito P450s, members of the CYP6Zs have been frequently associated with pyrethroid resistance [15,20–22]; however, this is not supported by functional studies as they do not appear to metabolize pyrethroids. Chiu et al. [23] showed that *An. gambiae* CYP6Z1 metabolized DDT, whereas pyrethroid metabolism was not mentioned. McLaughlin et al. [24] revealed that *An. gambiae* CYP6Z2 metabolized various substrates, but not pyrethroids. In *Ae. aegypti*, CYP6Z8 was found to be induced by insecticides and pollutants [22,25–28] and constitutively overtranscribed in pyrethroid-resistant populations [14,29]. This gene was also found preferentially transcribed in tissues classically involved in insecticide metabolism such as midgut and Malpighian tubules [27].

In this context, the present study aimed at characterizing *Ae. aegypti* CYP6Z8 substrate selectivity and its ability to metabolize insecticides. For this purpose, a yeast expression system allowing the co-expression of any mosquito microsomal P450 along with its associated CPR was developed. Using this system, we obtained a functional microsomal membrane complex of CYP6Z8 and *Ae. aegypti* CPR, which was used for *in vitro* metabolism assays. Our data indicate that although CYP6Z8 metabolizes various substrates, it is not capable of metabolizing most insecticides. However, our data reveal that CYP6Z8 and CYP6Z2, an anopheline orthologue of CYP6Z8, are likely to play a pivotal role in the clearance of pyrethroid insecticides via further catabolism of pyrethroid derivatives obtained by the action of carboxylesterases. This is significant from an operational vector control perspective as it is the first direct evidence that secondary metabolism of insecticides pyrethroids by P450s is linked to resistance. *In silico* 3D-modelling of substrate–enzyme interactions supports the involvement of other mosquito CYP6Zs in this process. The findings of the present study are discussed in regard to metabolic resistance mechanisms and detoxification pathways in mosquitoes.

## EXPERIMENTAL

### Materials, strains and media

Enzymes were purchased from New England Biolabs, oligonucleotides were purchased from Eurogentec, chemicals were purchased from Sigma–Aldrich and culture media were purchased from Euromedex. DNA sequencing was performed by Cogenics (Genome Express). *Ae. aegypti* mosquitoes from the Bora-Bora strain were bred under standard insectary conditions as described in Poupardin et al. [22]. Yeast *Saccharomyces cerevisiae* strain W303-1B (*MATα; leu2*, *his3*, *trp1*, *ade2-1*, *ura3*, *can^R^*, *cyr^+^*), henceforth known as W(N), represents the wild-type strain used for the construction of all other strains described in the present study and was kindly provided by Dr P. Urban and Dr D. Pompon (University of Toulouse, INSA-UPS-LISBP). SG(A)I synthetic minimum medium contained 20 g/l glucose (SGI), 1 g/Ll bactocasamino acids, 6.7 g/l yeast nitrogen base without amino acids and 40 mg/l DL-tryptophane. When required, 30 mg/l adenine was added (SGAI). N3 complete respiratory medium consisted of 10 g/l yeast extract, 10 g/l bactopeptone and 2% glycerol. YPDA complete media contained 20 g/l glucose, 10 g/l yeast extract, 10 g/l bactopeptone and 30 mg/l adenine. Repressive complete medium YPGE consisted of 5 g/l glucose, 10 g/l yeast extract, 10 g/l bactopeptone and 3% ethanol. Solid media consisted of media described above with an addition of 20 g/l agar. All culture media were sterilized at 121°C for 20 min before use.

### Cloning and heterologous expression of *Ae. aegypti* Cyt b5

The full cDNA sequence of *Ae. aegypti* Cyt b5 (VectorBase accession number AAEL010017) was amplified by PCR using high-fidelity Taq Expand™ (Roche Applied Science) and the following primers: forward, 5′-ATGTCGGAAGTGAAAACCTTCTCGC-3′, and reverse, 5′-GTTAGCAGGTTACTGAGTAAAGTAGAAC-3′. The amplification product was purified, cloned and sequenced. BamHI and EcoRI restriction sites were inserted by PCR using the following primers: forward, 5′-AAGGATCCAAAATGGCTGAAGTGAAAACC-3′ (start codon underlined), and reverse, 5′-GCCGAATTCTTTACTGAGTAAAGTAGAACC-3′. The fragment was purified, cloned into the pET22b expression vector (Novagen) and resequenced. Protein expression was performed in *Escherichia coli* Rosetta 2 (DE3) cells as described previously [30]. Cyt b5 was then purified on a HiPrep DEAE–Sepharose FF column (GE Healthcare) and eluted by a step gradient of NaCl. The fraction containing the purified Cyt b5 was then desalted using a HiPrep Desalting column (GE Healthcare) and concentrated using an Amicon Ultra YM10 filter unit (Millipore). High-molecular-mass contaminants were eliminated by using a HiLoad Superdex 75 column. Protein was concentrated again and purity was checked by SDS/PAGE. The Cyt b5 concentration was evaluated as described previously [31].

### Cloning of CYP6Z8 and *Ae. aegypti* CPR for expression in yeast

The full cDNA sequence encoding *Ae. aegypti* CYP6Z8 (VectorBase accession number AAEL009131-RA) was amplified by PCR using high-fidelity Taq Expand™ (Roche Applied Science) and the following primers: forward, 5′-GCAAAGAGTTCAAAATGGTCAT-3′, and reverse, 5′- GCACAAGTTCTCTATTCAGC-3′ (start and stop codons underlined). A similar procedure was used to amplify AeCPR (*Ae. aegypti* CPR; VectorBase accession number AAEL003349-RA) using the following primers: forward, 5′-ATGGACGCACAGACGGAAC-3′, and reverse, 5′-TTTCAGCGGGAGATGGATTAAC-3′ (start and stop codons underlined). Both amplicons were fully sequenced and the corresponding synthetic gene was synthesized by GeneCust with optimization for yeast expression (codon usage, mRNA structure, GC content, and insertion of BamH1 and EcoRI sites at 5′ and 3′ ends respectively).

### Construction of CYP6Z8 and CPR expression plasmids

CYP6Z8 and CPR synthetic genes were subcloned into yeast replicative pYeDP60 and yeast integrative pYeDP110 plasmids (kindly provided by Dr P. Urban) as described in Pompon et al. [32]. Both vectors can be propagated in *E. coli*, hold an expression cassette under a glucose-repressed and galactose-inducible *GAL10-CYC1* promoter and included an *URA3* marker (uracil auxotrophy complementation). The plasmid pYeDP60 also contains an adenine *ADE2* marker. Both plasmids were digested with BamHI and EcoRI and purified with the Gel Extraction Purification kit (Qiagen). *CYP6Z8* and *CPR* synthetic genes were ligated to pYeDP60 and pYeDP110 respectively using T4 DNA ligase and the ligation product was used to transform DH5α chemically competent *E. coli*. Positive colonies were detected by PCR. Plasmids containing CYP6Z8 and CPR constructs were purified, double-digested and sequenced. Expression plasmids containing synthetic genes encoding CYP6Z8 and CPR were called pYeDP60-6Z8 and pYeDP110-CPR.

### Stable integration of *Ae*CPR into the yeast genome

The W(AeR) strain was obtained by stable integration of the AeCPR under the control of the *GAL10-CYC1* promoter into the W(N) genome. This genome integration was performed by disrupting the yeast *CPR* gene. Briefly, pYeDP110-CPR was first linearized by NotI and purified using a gel-extraction kit. Then, W(N) yeast were transformed as described previously using the lithium acetate/single-strand carrier DNA/PEG method [33] and spread on SGAI-agar plates. Positive colonies were streaked individually on SGAI-agar plates, allowed to grow, and then streaked again on N3-agar plates to ensure they can grow in the absence of glucose (rho+ phenotype). Positive colonies were checked by PCR to ensure the integrity of the recombined genomic locus.

### Expression of CYP6Z8 in the W(AeR) yeast strain

W(AeR) yeast were transformed as described previously [33] by pYeDP60-6Z8. Positive colonies were streaked on SGI-agar plates and N3-agar plates and then checked by PCR to ensure the presence of the *CPR* gene and pYeDP60-6Z8 plasmid. Afterwards, 30 ml of stationary-phase SGI culture were used to inoculate 500 ml of YPGE and the culture was allowed to grow at 30°C for 48 h. When a *D*_600_ of 4 was reached, 20 g/l galactose was added to the culture to induce CPR and CYP6Z8 expression. The culture was further allowed to grow at 30°C for 7 h with horizontal agitation at 140 rev./min before microsome extraction.

### Preparation of yeast CYP6Z8 microsomes

The culture was first centrifuged at 2000 ***g*** for 18 min and the pellet washed with 1 ml of TEK [50 mM Tris/HCl (pH 7.4), 1 mM EDTA and 100 mM KCl] per 0.5 g of cells. The pellet was resuspended in 10 ml of TES [50 mM Tris/HCl (pH 7.4), 1 mM EDTA, 0.6 M sorbitol, 1 mM PMSF and 1 mM DTT] and glass beads were added. Yeast cells were broken by vortex-mixing five times for 30 s at 4°C. TES (5 ml) was added to wash the beads and the supernatant was transferred into a clean tube. The beads were washed again twice, the supernatants pooled and centrifuged at 10000 ***g*** for 10 min. The supernatant was further ultracentrifuged at 27000 rev./min (rotor SW 28, Beckman Coulter) for 1 h. The pelleted microsomes containing CYP6Z8 and CPR were resuspended in 500 μl of TEG [50 mM Tris/HCl (pH 7.4), 1 mM EDTA and 30% glycerol] per 500 ml of yeast culture, aliquoted and stored at −80°C until use [32].

### Quantification of CPR and CYP6Z8 in yeast microsomes

Microsomal protein concentration was determined using the Bradford method [34]. Specific content in P450s was measured from the reduced carbon monoxide difference spectra according to Omura and Sato [35] on both W(AeR)-CYP6Z8 microsomes and W(AeR) microsomes. CPR activities were measured in W(AeR)-CYP6Z8, W(AeR) and W(N) microsomes by following the reduction of cytochrome *c* at 550 nm, in the presence of NADPH [36].

### Activity of CYP6Z8 microsomes against standard P450 substrates

Four resorufin ethers (methoxyresorufin, ethoxyresorufin, pentoxyresorufin and benzyloxyresorufin; Sigma–Aldrich) were tested as fluorogenic substrates against W(AeR)-CYP6Z8 microsomes. For each sample, 5 pmol of P450 in a total reaction volume of 200 μl was added to 0.1 M phosphate buffer (pH 7.4) containing 5 μM substrate, 0.1 mM NADPH and an electron regeneration system (3 mM glucose 6-phosphate and 0.4 unit of glucose-6-phosphate dehydrogenase), and incubated at 30°C for 60 min. The production of resorufin was monitored by measuring fluorescence at 537 nm excitation and 587 nm emission with a Varioskan Flash Multimode Reader (Thermo Fisher Scientific). A standard curve of resorufin (Sigma–Aldrich) was used to calculate product formation rate. 7-Ethoxycoumarin O-de-ethylation was monitored in a similar manner. The reaction mix was identical as above, except that 8 pmol of P450 was used in a final volume of 100 μl. After 10, 30 and 60 min, the reaction was stopped by adding 100 μl of 50:50 (v/v) glycine/ethanol buffer and the production of 7-OH (7-hydroxycoumarin) was quantified by measuring the fluorescence at 380 nm excitation and 460 nm in comparison with an 7-OH standard (Sigma–Aldrich). The effect of the presence of Cyt b5 on CYP6Z8 activity was assessed by supplementing the incubation reaction with 2.5–50 pmol of Cyt b5. For the determination of kinetic parameters, the substrate concentration ranged from 0 to 10 μM and the incubation time was set to 15 min.

### *In vitro* metabolism assays with non-fluorescent substrates

*In vitro* incubations contained 10 μM substrate, 50 pmol of *Ae. aegypti* CYP6Z8 in W(aeR) microsomes or AgCYP6Z2 in *E. coli* membranes [24] when indicated, 5 mM MgCl_2_, 0.1 mM NADPH and the electron regenerative system (see above) for a total reaction volume of 100 μl. Control experiments consisted of omitting the NADPH regeneration system. After incubation at 30°C, the reaction was stopped by adding 100 μl of acetonitrile, shaking and incubation for 20 min and then centrifugation for 5 min at 16000 ***g*** to pellet microsomal proteins. The supernatant was then transferred into ultraclean glass vials and analysed by reverse-phase HPLC. The effect of the presence of Cyt b5 on CYP6Z8 activity was assessed by supplementing incubation mixtures with 80 pmol (8 equivalents) of purified *Ae*. *aegypti* Cyt b5.

Insecticide metabolism was monitored by reverse-phase HPLC on an Agilent 1260 HPLC system equipped with a Nucleodur C_18_ Polartec 250 mm×4.6 mm 3μ column (Macherey-Nagel) and a multi-wavelength photodiode array. Insecticides and potential metabolites were eluted with a gradient from 20 to 100% acetonitrile in water containing 0.1% TFA (trifluoroacetic acid) for 20 min, followed by a plateau at 100% for 4 min. For imidacloprid, a gradient from 10 to 50% acetonitrile in 20 min was used. Elution times of each compound and wavelengths used for their detection are shown in Table 1.

**Table 1:** Metabolism of various xenobiotics by CYP6Z8 Turnover is measured as pmol of substrate depleted/min per pmol of P450. ND, not detected.

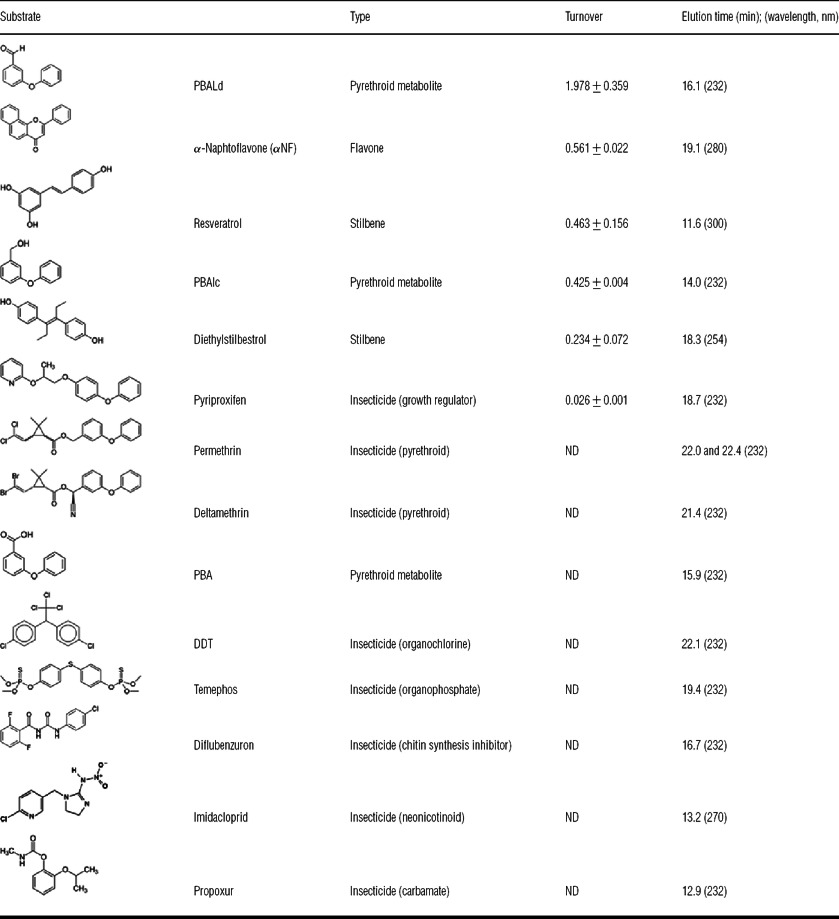

### MS identification of PBAlc (3-phenoxybenzoic alcohol) metabolites

The LC–MS/MS analysis was performed on an Agilent 1100 HPLC coupled to a Bruker Esquire 3000+ Ion Trap mass spectrometer (Bruker Daltonics) in a positive mode (ESI+) under the following conditions: nebulizer gas 11 p.s.i. (N2), drying gas 8 l/min, drying temperature 350°C, HV capillary 2000 V, HV End Plate Offset −500 V, capillary exit 103 V and skimmer 40 V.

### *In vivo* metabolism assays

The ability of *Ae. Aegypti* third instar larvae to metabolize PBAlc and excrete metabolites was assayed by exposing third-stage larvae for 24 h to 10 μM PBAlc. In total, 25 larvae were exposed to 250 μl of PBAlc (10 μM) in open microcentrifuge tubes for 24 h under standard insectary conditions (26°C, 14 h/10 h light/dark period, 80% relative humidity). Controls consisted of a PBAlc solution without larvae and a PBAlc solution with larvae previously killed by heating at 95°C for 10 min. After 24 h, reactions were flash-frozen in liquid nitrogen, thawed and centrifuged at 13000 ***g*** for 1 min. Supernatant was then transferred into HPLC vials containing an equal volume of acetonitrile and subjected to HPLC analysis as described above to quantify remaining PBAlc and excreted metabolites.

### Induction of *CYP6Z8* by hydrolysed pyrethroid metabolites

The induction of *CYP6Z8* by PBAlc, PBAld (3-phenoxybenzaldehyde) and PBA (3-phenoxybenzoic acid) was investigated by exposing third stage larvae for 24 h to 25, 250 and 2500 nM of each compound and measuring the *CYP6Z8* transcription level by reverse transcription followed by quantitative real-time PCR (RT-qPCR). Experimental procedures used for RNA extractions, reverse transcription, real-time PCR and primers used for qPCR are described in Poupardin et al. [27]. Data analysis was performed according to the ΔΔ*C*_T_ method taking into account PCR efficiency [37] and using the housekeeping gene encoding the ribosomal protein L8 (*AeRPL8*, GenBank® accession no DQ440262). Three independent biological replicates using different egg batches were performed and results were expressed as the mean transcription ratio relative to controls (unexposed larvae). Transcription data were computed by using a Mann–Whitney test on transcription ratios (*n*=3). Genes were considered significantly overtranscribed compared with controls when the mean transcription ratio was superior to 1.5 and the Mann–Whitney *P* value was <0.05.

### *In silico* substrate docking 3D modelling

The molecular models of CYP6Z8 and other CYP6Zs were created based on the crystal structure of CYP3A4 ([38]; PDB code 1TQN), currently the most homologous protein with a known structure, with 28% identity. Docking studies were carried out using GOLD v3.1 with the ChemScore scoring function [39] and an active site radius of 20 Å (1 Å=0.1 nm). Ligand structures were obtained from ChemIDPlus (http://chem.sis.nlm.nih.gov/chemidplus/). In total 50 binding modes were obtained for deltamethrin, PBAlc, PBAld and PBA. Figures were prepared using PyMOL (http://www.pymol.org).

## RESULTS

### CYP6Z8 sequence analysis

*Ae. aegypti CYP6Z8* was cloned from cDNA (Bora-Bora strain) and fully sequenced (*CYP6Z8v1*, GenBank® accession number JQ970488). Sequence analysis revealed 49 nucleotide variations compared with the genome sequence (gene AAEL009131, Liverpool strain, AaegL1.2 genset) (Supplementary Figure S1 at http://www.biochemj.org/bj/455/bj4550075add.htm). Of these, 13 were non-synonymous, leading to 97.14% identity with AAEL009131-PA (Supplementary Figure S2 at http://www.biochemj.org/bj/455/bj4550075add.htm). Only one non-synonymous variation was located within SRS (substrate recognition site) regions. This variation (C855A), leading to the replacement of a phenylalanine by a leucine at position 285 is not likely to affect substrate-binding properties. Indeed, structural models showed that this amino acid does not project into the active site and does not affect its dimensions. In addition, this variation was not predicted to affect the I helix position or substrate access channels (results not shown).

### Creating a yeast strain overexpressing mosquito CPR

The W(N) yeast strain was submitted to homologous recombination with the integrative plasmid pYeDP110-CPR to obtain the W(AeR) strain which overproduces AeCPR in the presence of galactose (*GAL* promoter). PCR with specific primers confirmed the stable replacement of the yeast *CPR* gene by the insert containing the *GAL* promoter and *AeCPR* gene. Microsomes extracted from galactose-induced W(AeR) cultures showed a significant overexpression of CPR activity compared with the W(N) strain (164±49 nmol of reduced cytochrome *c*/min per mg of protein compared with 32±13 nmol/min per mg of protein).

### Co-expression of recombinant CYP6Z8 and CPR in yeast

**Figure 1:**
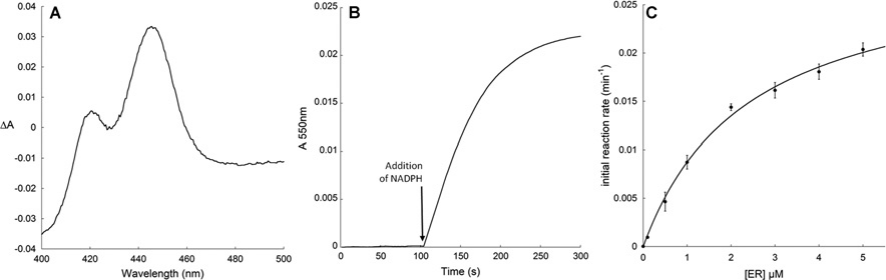
Biochemical characterization of yeast W(AeR)-CYP6Z8 microsomes (**A**) Fe^2+^-CO compared with Fe^2+^ difference spectrum. The spectrum was obtained from 1 mg of microsomal proteins containing 494 pmol of P450. (**B**) CPR activity of W(AeR) microsomes. Activity was quantified by monitoring the amount of reduced cytochrome *c* at 550 nm in the presence of NADPH and the NADPH regenerating system. (**C**) Michaelis–Menten plot of CYP6Z8–ethoxyresorufin (ER) kinetic parameters.

An average of 17 mg/l W(AeR)-CYP6Z8 microsomal proteins was obtained. The reduced CO-difference spectrum of W(AeR)-CYP6Z8 microsomes had a characteristic maximum absorption peak at 448 nm (Figure 1A). A minor peak was also observed at 420 nm (incorrectly folded protein), which increased to the detriment of the 450 nm peak for longer induction times, suggesting that CYP6Z8 is moderately stable. The average P450 concentration was 195±51 pmol/mg. Endogenous expression of yeast P450s was not detectable in W(N) or W(AeR) microsomes. Finally, the expression of AeCPR ranged from 97 to 209 nmol of reduced cytochrome *c*/min (Figure 1B) and was not significantly affected by the co-expression of CYP6Z8.

### CYP6Z8 activity against standard P450 fluorescent substrates

W(AeR) microsomes expressing only AeCPR did not show any significant activity against any P450 fluorescent substrate, confirming the very low expression of endogenous yeast P450s under these conditions. W(AeR)-CYP6Z8 microsomes were able to metabolize fluorescent substrates at different rates (Table 2 and Figure 1C). Determining apparent kinetic parameters, *K*_m_ and *V*_max_, for each fluorescent substrate showed that CYP6Z8 metabolized preferentially ethoxyresorufin and benzyloxyresorufin (*k*_cat_/*K*_m_ ratios of 0.49 and 0.74 respectively), whereas pentoxyresorufin and 7-ethoxycoumarin were metabolized at much lower rates (*k*_cat_/*K*_m_ ratios of 0.0094 and 0.17 respectively). Apparent kinetic parameters for methoxyresorufin could not be determined due to a non-Michaelian behaviour of this substrate. Although Cyt b5 integration into W(AeR)-CYP6Z8 microsomes and its interaction with the P450 were observed (results not shown), supplementing the reaction with recombinant Cyt b5 did not lead to a significant increase in activity for any of the substrates tested.

**Table 2:** CYP6Z8 specific activity against standard fluorescent P450 substrates ND, not determined because of non Michaelis–Menten behaviour.

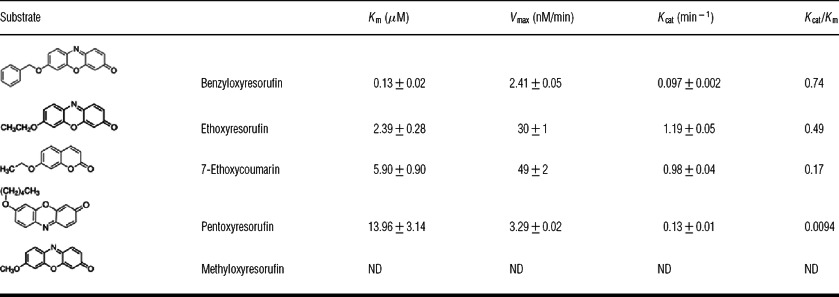

### CYP6Z8 activity against insecticides and other xenobiotics

Metabolism of insecticides from various chemical classes was assayed with W(AeR)-CYP6Z8 microsomes in the presence or absence of NADPH and the NADPH regenerating system. The degradation of the substrate and appearance of metabolites were monitored by reverse-phase HPLC (Table 1). The insecticides permethrin, deltamethrin, DDT, temephos, diflubenzuron, imidacloprid and propoxur were not significantly metabolized by CYP6Z8. However, CYP6Z8 metabolized different natural and synthetic xenobiotics such as α-naphthoflavone, resveratrol, diethylstilbestrol and to a lesser extent the insecticide pyriproxifen. As for fluorescent substrates, the presence of Cyt b5 did not affect CYP6Z8 substrate specificity and turnover (results not shown).

**Figure 2:**
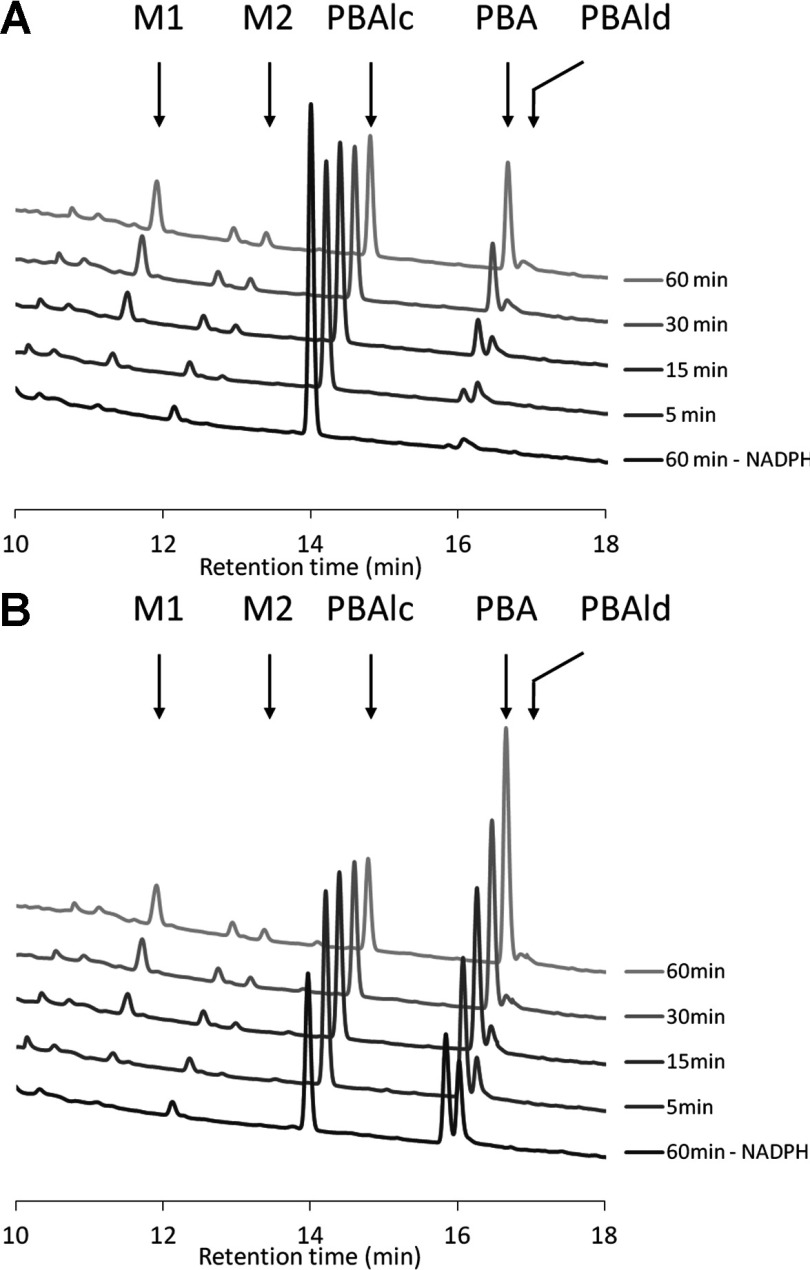
Analysis of PBAlc and PBAld metabolism by CYP6Z8 HPLC chromatograms showing the time course of PBAlc (**A**) and PBAld (**B**) metabolism by W(AeR)-CYP6Z8 microsomes. The lowest off-set corresponds to the negative control (−NADPH) followed by reactions in the presence of NADPH stopped after 5, 15, 30 and 60 min. PBAlc, PBAld, PBA and metabolite peaks are indicated by arrows on the 60 min off-set.

As pyrethroid catabolism may involve carboxylesterase-mediated hydrolysis as a first step, the conversion of intermediate metabolites, namely PBAlc and PBAld was also examined. When PBAlc was used as a substrate (Figure 2A), no significant metabolism occurred in the absence of NADPH and the NADPH regenerating system. In the presence of NADPH, PBAlc was metabolized by CYP6Z8 at a rate of 0.42 pmol/min per pmol of P450. PBAlc turnover was not significantly affected by the presence of Cyt b5 (results not shown). Several metabolites were produced by CYP6Z8 including PBA [*R*_t_ (retention time) 15.8 min], PBAld (*R*_t_ 16 min) and two more hydrophilic metabolites, M1 and M2 (*R*_t_ 11.1 min and 12.6 min respectively). PBA accumulated gradually and was the major metabolite after 60 min incubation, followed by the M1 metabolite. PBAld never accumulated to a large extent; its proportion increased progressively over 15 min before decreasing for longer incubation times, suggesting the further conversion of this metabolite. Finally, a minor M2 metabolite gradually appeared during incubation. Kinetic parameters of PBAlc metabolism were *K*_m_=15.1±2.9 μM, *V*_max_=33.4±1.5 pmol/min, *k*_cat_=0.66±0.03 min^−1^ and *k*_cat_/*K*_m_=0.046.

When PBAld was used as a substrate (Figure 2B), a significant conversion into PBAlc occurred in the absence of CYP6Z8, suggesting that this reduction is CYP-independent. Conversion into PBA strongly increased in the presence of CYP6Z8 and NADPH (1.98 pmol/min per pmol of P450), confirming that CYP6Z8 also metabolizes PBAld. PBAlc remained a significant metabolite over time, indicating equilibrium between oxidation and reduction reactions. As for PBAlc, a minor production of M1 and M2 metabolites was noticed and PBAld turnover was not significantly affected by the presence of Cyt b5 (results not shown). Finally, no PBA metabolism occurred in the presence of NADPH, indicating that this compound is not further metabolized by CYP6Z8.

The ability of *An. gambiae* CYP6Z2, an anopheline orthologue of CYP6Z8, to metabolize PBAlc and PBAld was also investigated. These experiments performed with *E. coli* recombinant CYP6Z2 [24] under identical conditions demonstrated the ability of CYP6Z2 to metabolize both PBAlc and PBAld, producing PBA together with minor M1 and M2 metabolites in the same way as CYP6Z8 (Supplementary Figure S3 at http://www.biochemj.org/bj/455/bj4550075add.htm).

### Identification of metabolites

Positive-mode electrospray LC–MS detected PBAlc, PBAld, PBA, M1 and M2 at 183, 199, 215, 200 and 228 *m*/*z* respectively. PBAlc was detected 18 units below its theoretical mass (201 *m*/*z*), indicating its dehydration during LC-MS (Supplementary Table S1 at http://www.biochemj.org/bj/455/bj4550075add.htm). MS analyses did not allow the full identification of M1 and M2 metabolites. However, M1 and M2 metabolites did not appear to result from the direct or sequential hydroxylation of PBAlc or PBAld as no +16 *m*/*z* were observed. In addition, LC–MS *m*/*z* obtained for M1 and M2 did not match with any expected metabolite structure.

MS/MS analyses by positive-mode electrospray fragmentation detected various fragmentation ions associated with each compound (Supplementary Table S2 at http://www.biochemj.org/bj/455/bj4550075add.htm). The fragment shared by PBAld and PBA (171 *m*/*z*) is likely to correspond to diphenylether. This fragment could not be identified from M1 or M2, suggesting that these two metabolites rather originate from PBAlc. In addition, among all fragments, one was shared between PBAlc and M1 (155 *m*/*z*), suggesting a common backbone of these two compounds. This fragment was not shared with M2. Moreover, replacing H_2_O/0.1% TFA (pH 2.0) by 10 mM Tris buffer (pH 7.5) for HPLC analysis resulted in the shift of PBA and PBAld peaks (results not shown), whereas PBAlc, M1 and M2 peaks were not affected (no change in signal intensity and *R*_t_), suggesting that M1 and M2 do not possess any pH-dependent function, unlike PBAld or PBA.

### *In silico* 3D modelling of CYP6Z–substrate interactions

**Figure 3:**
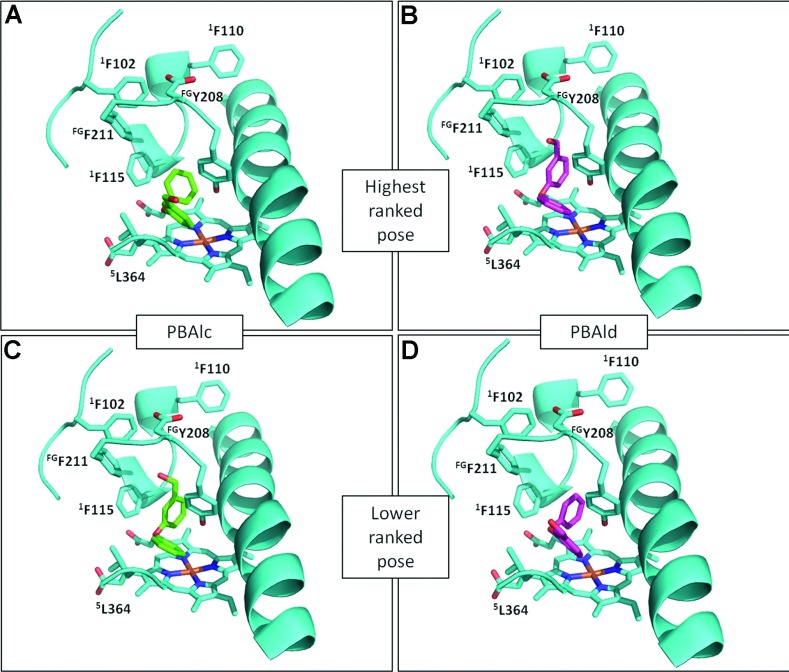
Predicted binding modes of PBAlc and PBAld in the CYP6Z8 model PBAlc (green) and PBAld (purple) binding are shown for the highest-ranked poses (**A** and **B**) and lower-ranked poses (**C** and **D**). Residues that project into the active site are labelled. Superscript text refers to amino acids belonging to SRS1 (^1^), FG loop (^FG^) and SRS5 (^5^) regions.

**Figure 4:**
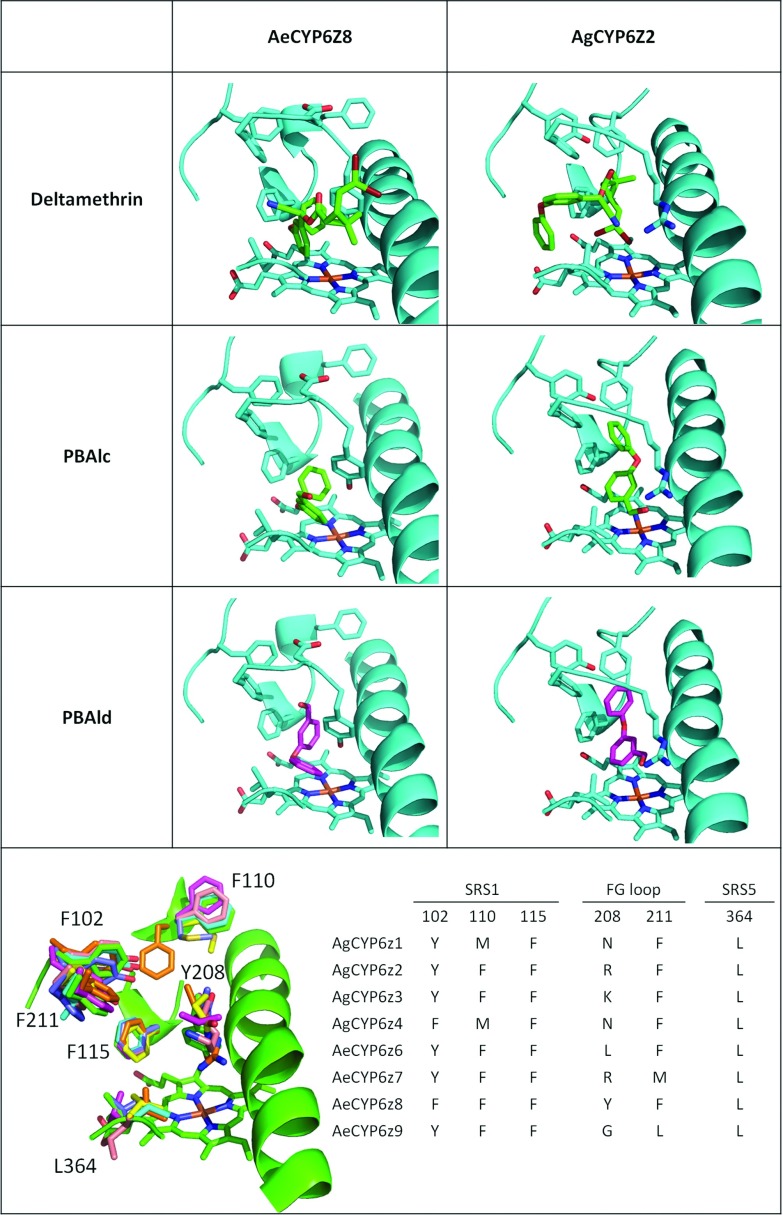
Molecular models of CYP6Zs Top panel: comparison of deltamethrin, PBAlc and PBAld docking in AeCYP6Z8 and AgCYP6Z2 (highest-ranked poses). Bottom panel: alignment of AeCYP6Z8 (green), AeCYP6Z9 (blue), AeCYP6Z6 (purple), AgCYP6Z4 (yellow), AgCYP6Z3 (pink), AgCYP6Z1 (dark blue) and AgCYP6Z2 (orange). The table shows a comparison of residues projecting into the active site. Residues are labelled according to the position on CYP6Z8. Ae, *Ae. aegypti*; Ag, *An. gambiae*.

In order to better understand why pyrethroids and their metabolites were metabolized or not by CYP6Zs, 3D models were built and the docking modes of PBAlc, PBAld, PBA and deltamethrin were predicted *in silico*. In CYP6Z8 and CYP6Z2, PBAld and PBAlc bound in two alternative modes that allow metabolism either on the phenyl or benzyl rings (Figure 3), consistent with experimental findings showing two metabolites. Given the phenyl ring structure of PBAld and PBAlc, interactions with aromatic side-chain amino acids are of potential interest in substrate binding and metabolism. A cluster of aromatic side chains are predicted in the active site of CYP6Z8, Phe^102^, Phe^110^, Phe^115^, Tyr^208^ and Phe^211^, of which Phe^115^ and Tyr^208^ appear to be positioned as clamps above the haem iron atom, thus potentially key residues in substrate positioning for catalysis (Figure 3). Orientation in two binding modes is predicted, consistent with the two products observed (PBAld and PBA). Despite their lack of deltamethrin metabolism, both CYP6Z8 and CYP6Z2 produced their highest binding scores with this compound (~42 kJ/mol) (Supplementary Table S3 at http://www.biochemj.org/bj/455/bj4550075add.htm). However metabolism appears to be prevented in CYP6Z8 by internal clashes with Tyr^208^, and in CYP6Z2 by residues close to the haem (Leu^365^ and Phe^115^). Steric hindrance of PBA does not appear to prevent metabolism since productive pose are predicted in CYP6Z8. However, the low binding score points to a low affinity for this molecule (Supplementary Table S3). Examination of structural models of other *Ae. aegypti* and *An. gambiae* CYP6Zs indicates they may all have the capacity to metabolize PBAlc and PBAld and produce similar metabolites (Figure 4).

### *In vivo* assays and regulation of CYP6Z8 expression by pyrethroid metabolites

**Figure 5:**
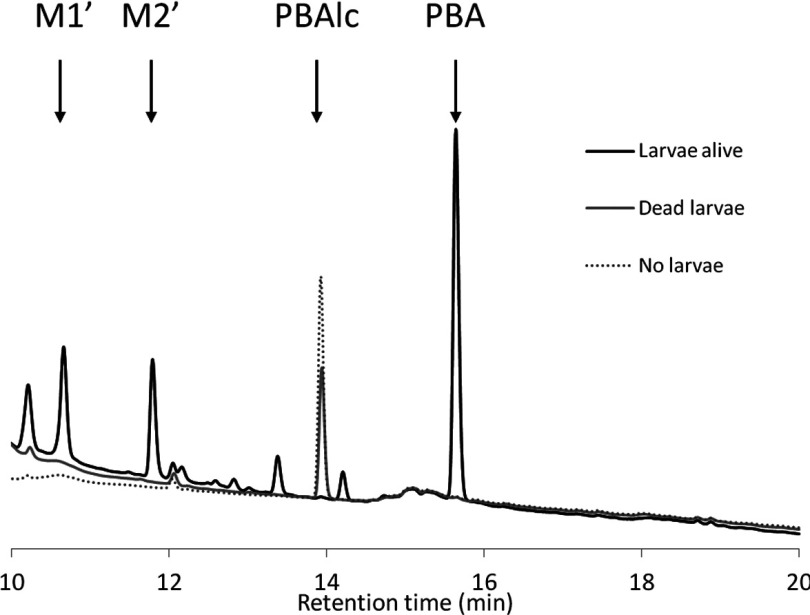
*In vivo* metabolism of PBAlc HPLC chromatograms showing excreted metabolites after exposing *Ae. aegypti* larvae alive or dead for 24 h to 2 mg/l PBAlc. PBAlc and main metabolites are indicated by arrows.

Incubating alive *Ae. aegypti* third instar larvae with PBAlc for 24 h revealed a concomitant decrease in PBAlc in the growing medium and an increase in PBA together with other minor metabolites, suggesting that the PBA produced is directly excreted by larvae without further modification (Figure 5).

In order to assess whether *CYP6Z8* transcription is regulated by its substrates and/or products, larvae were exposed for 24 h to various concentrations of PBAlc, PBAld and PBA. The transcription levels of *CYP6Z8* compared with unexposed larvae were then compared using qPCR (Supplementary Figure S4 at http://www.biochemj.org/bj/455/bj4550075add.htm). *CYP6Z8* transcription was significantly induced by all compounds (2.0–3.4-fold) with a moderate dose-dependent effect.

## DISCUSSION

The present study aimed at co-expressing *Ae. aegypti* CYP6Z8 and AeCPR in an heterologous system, characterizing its activity towards known P450 substrates and investigating its role in the metabolism of insecticides.

CYP6Z8 sequence analysis revealed several variations in the Bora-Bora strain compared with the genome sequence (Liverpool strain, VectorBase AaegL1.2 genset). In total 49 nucleotides variations, including 13 variations leading to amino acid changes, were found, confirming the high polymorphism of mosquito CYPs. These non-synonymous variations did not occur in the conserved P450 signature motifs [12]. Only one amino acid change occurred in an SRS at a position not strictly conserved among CYP6Zs. As confirmed by 3D modelling, this replacement (F285L) occurring in the oxygen-binding motif (SRS4) is likely not to affect CYP6Z8 substrate specificity, as physicochemical properties of these two amino acids are similar (Figure 4).

The yeast P450 expression system developed by Pompon et al. [32] was chosen to create a novel genetically modified yeast strain allowing the co-expression of any mosquito P450 together with its CPR. Such a system has been successfully used to express and characterize various P450s from human, plants and fungi [32,40–42]. The results of the present study show that replacing yeast CPR by mosquito CPR under a galactose-inducible promoter enables the W(AeR) strain to overexpress mosquito CPR at a high level. Transforming the W(AeR) strain with an expression plasmid carrying the *CYP6Z8* gene under the same promoter allowed us to produce a functional mosquito CPR–P450 membrane system. As CPR is highly conserved in mosquitoes, this new tool should allow the functional expression of any microsomal mosquito P450. This was recently confirmed by the successful expression of functional *An. gambiae* P450s using the W(AeR) yeast strain (A. Chandor-Proust, M. Paine and J.-P. David, unpublished work). To our knowledge, this is the first time a mosquito P450 has been successfully expressed in yeast, along with its redox partner and without protein sequence modification. Although *E. coli* is commonly used to express P450s and has higher yields, some eukaryote P450s are intractable for expression. The yeast system thereby provides a useful alternative method with the advantage of having eukaryotic cellular and redox machinery plus organelle structure appropriate for the translation of eukaryotic P450s.

The results of the present study clearly indicate that our recombinant CYP6Z8 is functional and able to metabolize various standard P450 substrates. Kinetic data suggest that benzyloxyresorufin is preferred, followed by ethoxyresorufin. Supplementing reactions with purified *Ae. aegypti* Cyt b5 did not affect CYP6Z8 substrate specificity and activity. Whether CYP6Z8 does not strongly interact with Cyt b5 or the co-expression of AeCPR and CYP6Z8 produces such a high P450/CPR ratio making the presence of an extra electron donor, such as Cyt b5, not necessary requires further investigation [43].

Then, the ability of CYP6Z8 to metabolize various xenobiotics, including insecticides, was investigated. As for *An. gambiae* CYP6Z2, CYP6Z8 did not metabolize permethrin and deltamethrin [23,24]. In contrast with CYP6Z1, CYP6Z8 was not capable of metabolizing DDT either. However, CYP6Z8 metabolized α-naphthoflavone and the stilbene resveratrol, as did CYP6Z2 [24].

**Figure 6:**
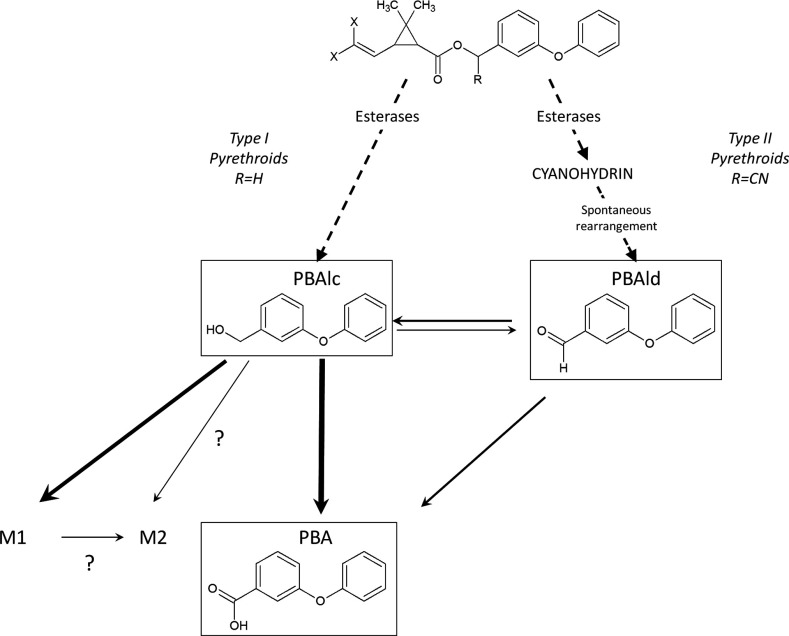
Proposed role of CYP6Z8 in pyrethroid metabolism Broken arrows indicates a first attack by carboxylesterases [52]. Solid arrows indicate CYP6Z8 NADPH-dependent transformations. Arrow width is proportional to the initial reaction rate of metabolite production. Hypothetical reactions forming the M2 metabolite are indicated by question marks. In the generic representation of pyrethroids, X represents a halogenated substituent and R is either a H (type I pyrethroid) or CN (type II pyrethroid) group.

In mammals it has been demonstrated that pyrethroids can be hydrolysed by carboxylesterases leading to the production of PBAlc and PBAld and that these metabolites can be further processed by P450s into PBA [44–46]. Recently, *in vitro* metabolism assays with microsomes extracted from *Ae. aegypti* larvae suggested that this detoxification pathway occurs in mosquitoes [47]. Because CYP6Zs are frequently overtranscribed in mosquito populations resistant and/or exposed to pyrethroids, the ability of CYP6Z8 to metabolize common pyrethroid metabolites was investigated further. Our results clearly demonstrate that CYP6Z8 is capable of metabolizing PBAlc into PBA, with PBAld being a transitory metabolite (Figure 6). Similarly, we showed that its *An. gambiae* orthologue CYP6Z2 can also metabolize PBAlc in the same manner, supporting the functional orthology of these two mosquito P450s. To our knowledge, CYP6Z8 and CYP6Z2 are the first insect P450s shown to metabolize pyrethroid metabolites generated by carboxylesterase hydrolysis.

Two minor more hydrophilic metabolites (M1 and M2) were also detected. Examination of M1 and M2 metabolites by LC–MS and MS/MS did not allow the absolute identification of their chemical structure. The behaviour of PBAlc in positive-mode electrospray was not trivial as the parental ions (183 *m*/*z*) did not correspond to the theoretical mass (201 *m*/*z*), but suggested its dehydration. Despite this, no direct additions of alcohol function (+16 *m*/*z*) were observed for M1 and M2, suggesting that these compounds are not simply hydroxylated. Previous studies have shown that the direct attack of pyrethroids by P450s often produces 4′-hydroxylation in insects [17,48]. The results of the present study suggest that the attack of pyrethroid metabolites generated by esterase hydrolysis by CYP6Zs is different.

Because PBA is not metabolized by CYP6Z8, M1 and M2 can only represent metabolites of PBAlc or PBAld. Common fragmentation ions were found between M1 and PBAlc, suggesting a similar backbone. In addition, M1 and M2 HPLC *R*_t_ were not affected by pH variations as opposed to PBAld and PBA, confirming that they do not carry acid or aldehyde functions. Overall, these results support the hypothesis of M1 originating from PBAlc and M2 being a secondary metabolite of M1 (Figure 6). Finally, *in vivo* experiments revealed that PBA is the major metabolite produced and is excreted from mosquitoes without further modification. In contrast, M1 and M2 metabolites were not excreted from mosquitoes. Instead, two more polar metabolites (M1′ and M2′ in Figure 5) separated by a comparable Δ*R*_t_ were observed, suggesting that M1 and M2 are further processed before being excreted.

*In silico* 3D modelling of CYP6Z–substrate interactions confirmed our experimental findings on CYP6Z8 and CYP6Z2 with good binding scores and limited clashes of pyrethroid metabolites in the active site. Comparing 3D models between various *Aedes* and *Anopheles* CYP6Zs supports the capacity of other mosquito CYP6Zs to metabolize these and produce similar metabolites (Figure 4). The inability of CYP6Z8 and CYP6Z2 to metabolize deltamethrin was supported by 3D models showing good binding scores but high clashes between active-site residues and substrate or within substrate itself. Bulky residues in the FG loop and SRS1 and SRS5 of mosquito CYP6Zs are probably preventing the metabolism of large pyrethroids. Indeed, while known pyrethroid metabolizers such as CYP6M2, CYP6B8 and CYP3A1 may require aromatic residues in the active site at position Phe^115^ to π-stack with the phenyl ring, and/or Phe^102^ to bind with the benzyl ring [17,49,50], the extensive network of aromatic residues in the CYP6Z subfamily may restrict the binding of the large pyrethroids, but stabilize the binding of smaller compounds such as PBAlc or PBAld.

Overall, the results of the present study lift the veil on the pivotal role of CYP6Z8 and CYP6Z2 in pyrethroid biodegradation in mosquitoes and clarify why they have frequently been associated with resistance [15,21,22,29,51], whereas their capacity to metabolize pyrethroid insecticides could not be validated [23,24]. This is the first direct evidence that secondary metabolism of insecticide pyrethroids by P450s is linked to resistance. Our results strongly support the role of these P450s following the action of carboxylesterases in order to clear mosquito body from these metabolites. Although hydrolysed pyrethroid metabolites such as PBAlc and PBAld are far less toxic than intact pyrethroids, their accumulation is likely to be detrimental to mosquitoes. Therefore the role of CYP6Zs in the mosquito response to pyrethroids is not negligible and the over-transcription of these genes in natural populations should be considered as supporting evidence of metabolic resistance.

Genes encoding carboxylesterases have been frequently found to be overtranscribed in pyrethroid-resistant populations and their role in pyrethroid biodegradation has been established *in vitro* [47]. However, to our knowledge, no particular mosquito carboxylesterase has yet been validated as a pyrethroid metabolizer. A better understanding of insecticide degradation pathways in mosquitoes will allow for the pinpointing of action points to develop new strategies to overcome resistance mechanisms. In this frame, estimating the relative importance of mosquito detoxification enzymes in resistance is also important, but not trivial. Any enzyme involved in the insecticide degradation pathway may have a different importance in the resistant phenotype depending on its expression profile, the step catalysed, its substrate specificity and turnover rate, and the toxicity and lipophilicity of the metabolites produced. This certainly represents the next research challenge for understanding how mosquitoes adjust their metabolism to resist insecticides and better manage these resistance mechanisms.

## Online data

Supplementary data
