# Supplementary material for: The central role of mosquito cytochrome P450 CYP6Zs in insecticide detoxification revealed by functional expression and structural modelling
Source: Biochem J. 2013 Sep 13;455(Pt 1):75–85. doi: 10.1042/BJ20130577 (PMC3778711; doi:10.1042/BJ20130577)
Supplement: Supplementary data [file bj4550075add.pdf]

BJ Metabolism

Biochem. J. (2013) **455**, 75–85 (Printed in Great Britain) doi:10.1042/BJ20130577

online data

SUPPLEMENTARY ONLINE DATA

The central role of mosquito cytochrome P450 CYP6Zs in insecticide detoxification revealed by functional expression and structural modelling

Alexia CHANDOR-PROUST*, Jaclyn BIBBY†, Myriam RÉGENT-KLOECKNER*, Jessica ROUX*, Emilie GUITTARD-CRILAT‡, Rodolphe POUPARDIN*§, Muhammad Asam RIAZ*‖, Mark PAINE§, Chantal DAUPHIN-VILLEMANT‡¶, Stéphane REYNAUD* and Jean-Philippe DAVID*[1]

*Laboratoire d'Ecologie Alpine (LECA), UMR 5553 CNRS, Université de Grenoble, Grenoble 38041, France, †Institute of Integrative Biology, University of Liverpool, Liverpool L69 7ZB, U.K., ‡Université Pierre et Marie Curie, CNRS, Paris 75005, France, §Liverpool School of Tropical Medicine, Liverpool L3 5QA, U.K., ‖Department of Agri-Entomology, University College of Agriculture, University of Sargodha, Sargodha, Pakistan, and ¶Department of Ecology and Evolution, University of Lausanne, Lausanne 1015, Switzerland

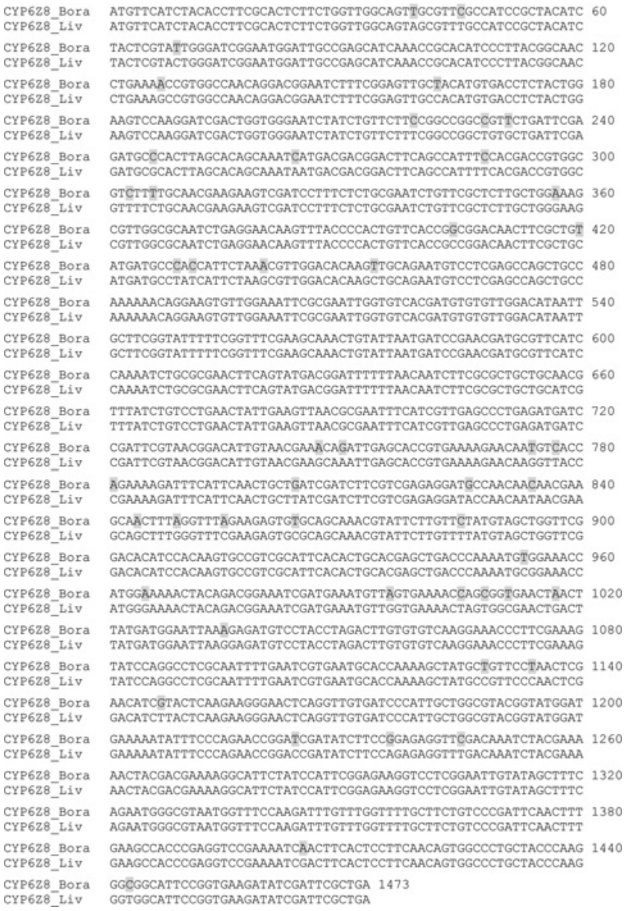

Figure S1 Alignment of cloned *CYP6Z8* cDNA compared with the genome sequence

Coding sequence cloned from Bora-Bora strain cDNA was compared with the cDNA sequence extracted from VectorBase (AaegL1.2 geneset, Liverpool strain). Differences are shaded.

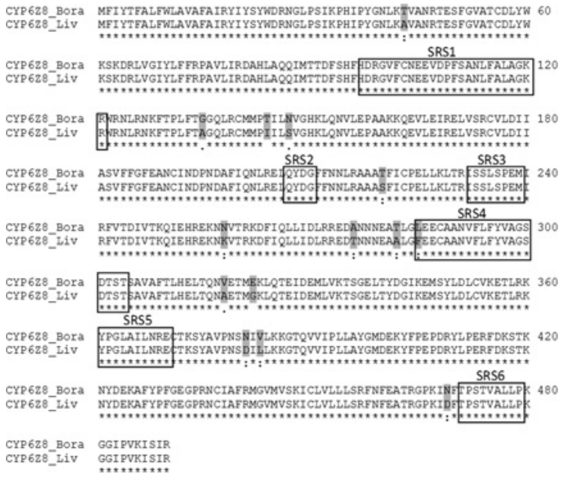

Figure S2 Alignment of *Ae. aegypti* CYP6Z8 protein sequences

Differences between CYP6Z8 obtained from the genome sequence (AaegL1.2 geneset, Liverpool strain) and CYP6Z8 cloned from the Bora-Bora strain are shaded. The degree of sequence conservation is indicated by standard motifs. Substrate-binding site regions are indicated by boxes.

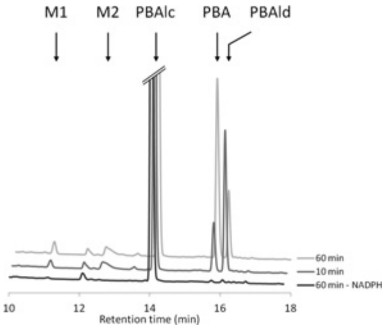

Figure S3 Analysis of PBAlc metabolism by *An. gambiae* CYP6Z2

HPLC chromatograms (off-set) showing the time course of PBAlc metabolism by AgCPR-CYP6Z2 membranes. The lowest off-set corresponds to the negative control (− NADPH) followed by reactions in the presence of NADPH stopped after 10 and 60 min. PBAlc, PBAld, PBA and metabolite peaks are indicated.

[1] To whom correspondence should be addressed (email jean-philippe.david@ujf-grenoble.fr).

© 2013 The Author(s)
The author(s) has paid for this article to be freely available under the terms of the Creative Commons Attribution Licence (CC-BY) (http://creativecommons.org/licenses/by/3.0/) which permits unrestricted use, distribution and reproduction in any medium, provided the original work is properly cited.

Biochemical Journal www.biochemj.org

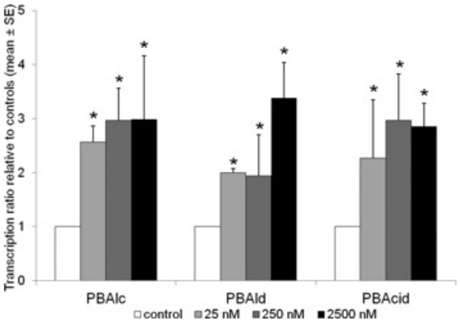

Figure S4 Induction of *CYP6Z8* by PBAlc, PBAld and PBA

Third-stage larvae were exposed for 24 h to three increasing doses of each compound. Transcription levels were measured by qPCR on pools of larvae from three independent replicates and expressed as means ± S.E.M. relative to controls (unexposed larvae). The significance of transcription ratios relative to controls were assessed by a Mann–Whitney test ($n = 3$). $^*P < 0.05$.

Table S1 LC–MS analysis of PBAlc and PBAld metabolites

Masses of $[M+H]^+$ ions were measured by HPLC coupled with MS (electrospray positive mode). Theoretical expected mass in this mode, dehydrated mass and observed mass are indicated. -, no dehydration is observed; ?, whether dehydration occurs or not is unknown.

Compound	Theoretical mass	Dehydration (−18)	Observed mass
PBAlc	201	183	183
PBAld	199	-	199
PBA	215	-	215
M1	Unknown	?	200
M2	Unknown	?	228

Table S2 MS/MS analysis of PBAlc and PBAld metabolites

Fragmentation ions observed in MS/MS (electrospray positive mode) are shown for each compound. Parental ions ($[M+H]^+$) are shown in bold. Common fragments are underlined.

Compound	Observed mass of fragmentation ions													
PBAlc							**183**					165	155	
PBAld					199						171			
PBA		**215**									171			
M1				**200**				182		172			155	
M2	**228**		211			193			175					151

Table S3 Docking binding scores

Binding scores of the best ranked poses (kJ/mol) of PBALc, PBAld, PBA and deltamethrin in members of the CYP6Z family. Ae, *Ae. aegypti*; Ag, *An. gambiae*.

Family	PBAlc	PBAld	PBA	Deltamethrin
AgCYP6Z1	34.49	34.64	31.99	37.24
AgCYP6Z2	37.09	36.46	33.43	42.69
AgCYP6Z3	36.73	36.18	33.69	43.4
AgCYP6Z4	34.22	32.17	31.55	42.76
AeCYP6Z6	34.25	34.43	30.59	45.24
AeCYP6Z8_bora	37.63	35.71	32.28	42.93
AeCYP6Z8_liv	36.34	36.42	31.97	42.55
AeCYP6Z9	31.15	30.6	28.68	41.1

Received 25 April 2013/10 July 2013; accepted 11 July 2013
Published as BJ Immediate Publication 11 July 2013, doi:10.1042/BJ20130577

© 2013 The Author(s)
The author(s) has paid for this article to be freely available under the terms of the Creative Commons Attribution Licence (CC-BY) (http://creativecommons.org/licenses/by/3.0/) which permits unrestricted use, distribution and reproduction in any medium, provided the original work is properly cited.
